# Triptans and CGRP blockade – impact on the cranial vasculature

**DOI:** 10.1186/s10194-017-0811-5

**Published:** 2017-10-10

**Authors:** Silvia Benemei, Francesca Cortese, Alejandro Labastida-Ramírez, Francesca Marchese, Lanfranco Pellesi, Michele Romoli, Anne Luise Vollesen, Christian Lampl, Messoud Ashina, On behalf of the School of Advanced Studies of the European Headache Federation (EHF-SAS)

**Affiliations:** 1 Health Sciences Department, University of Florence, and Headache Centre, Careggi University Hospital, Viale Pieraccini 6, 50134 Florence, Italy; 2 Department of Medico-Surgical Sciences and Biotechnologies, Sapienza University of Rome, Polo Pontino, Latina, Italy; 3 Dept Internal Medicine, Division of Vascular Pharmacology, Erasmus Medical Center, Rotterdam, The Netherlands; 4 Child Neuropsichiatry Unit, University of Palermo, Palermo, Italy; 5 Medical Toxicology Headache and Drug Abuse Center, University of Modena and Reggio Emilia, Modena, Italy; 6 Neurology Clinic, University Hospital of Perugia, Perugia, Italy; 7 Danish Headache Center and Department of Neurology, Rigshospitalet Glostrup, Faculty of Health and Medicl Sciences, University of Copenhagen, Copenhagen, Denmark; 8 Department of Neurogeriatric Medicine, Headache Medical Center Linz, Linz, Austria; 9 Danish Headache Center and Department of Neurology, Rigshospitalet Glostrup, Faculty of Health and Medical Sciences, University of Copenhagen, Copenhagen, Denmark

**Keywords:** Triptans, Calcitonin gene related peptide – CGRP, Anti-CGRP (receptor) monoclonal antibodies – mAbs, Middle meningeal artery, Middle cerebral arteries, Migraine models, Magnetic resonance angiography (MRA)

## Abstract

The trigeminovascular system plays a key role in the pathophysiology of migraine. The activation of the trigeminovascular system causes release of various neurotransmitters and neuropeptides, including serotonin and calcitonin gene-related peptide (CGRP), which modulate pain transmission and vascular tone. Thirty years after discovery of agonists for serotonin 5-HT_1B_ and 5-HT_1D_ receptors (triptans) and less than fifteen after the proof of concept of the gepant class of CGRP receptor antagonists, we are still a long way from understanding their precise site and mode of action in migraine. The effect on cranial vasculature is relevant, because all specific anti-migraine drugs and migraine pharmacological triggers may act in perivascular space. This review reports the effects of triptans and CGRP blocking molecules on cranial vasculature in humans, focusing on their specific relevance to migraine treatment.

## Keypoints

Triptans constrict extracerebral, but no intracerebral arteries, in healthy volunteers and migraine patients. The vasoconstrictor action of sumatriptan on extracerebral arteries could be relevant to relief migraine pain. However, sumatriptan also inhibits perivascular neurogenic inflammation and sensitization in animal models.

Gepants prevent CGRP-induced dilation of extracerebral arteries (e.g. middle meningeal and temporal arteries) in experimental human models.

Data on effect of anti-CGRP (receptor) monoclonal antibodies on cranial vasculature is still lacking. Importantly, preclinical models show their ability to inhibit CGRP-induced neurogenic vasodilation of the middle meningeal artery.

## Background

Over the last century, controversies have raised around the vascular, neural or neurovascular origin of migraine [1]. From Galen original conjecture [2], with a meningeal involvement in the throbbing pain, several centuries passed before Willis, in 1672, hinted for the first time at a “vascular hypothesis” of migraine [3]. Throughout the 1930s and early 1940s headache science has emerged from studies by Graham, Ray and Wolff, who reported head pain after in vivo stimulation of dural and cerebral arteries, hypothesizing perivascular space as the possible site of migraine pain [4–7]. Pial, dural and extracranial vessels are part of a trigeminovascular system, a functional pathway that, on one side, releases vasoactive neuropeptides from perivascular nerve fibers and, on the other, reacts to them with nociception and vasodilation [8]. Pursuing the vascular hypothesis, several pharmacological triggers (such as glyceryl trinitrate (GTN), calcitonin gene-related peptide (CGRP) and pituitary adenylate cyclase-activating peptide (PACAP-38) were found to induce attacks phenotypically indistinguishable from spontaneous migraine in migraine patients [9–11]. The fact that all migraine-provoking molecules are vasoactive and sumatriptan constricts arteries [12, 13], further granted a key role of cranial vasculature in migraine pathophysiology [14].

Cranial arteries dilation has been shown, with different techniques, in both provocation and spontaneous migraine studies. Since the early 1990s, ultrasonography has been used to measure blood flow velocity in intracranial arteries [15] and extracranial artery diameter [16] during migraine attacks. Blood flow velocity correlates to vessel autoregulation and reactivity. Moreover, if cerebral blood flow does not change during an attack, blood flow velocity may be a surrogate marker of artery diameter (i.e. decreased blood flow velocity means increased middle cerebral artery lumen) [17]. In the last decade, investigation techniques have moved from ultrasonography to magnetic resonance angiography (MRA), allowing researchers to directly measure artery circumference [18–22]. MRA studies reported modest artery dilation during attacks, which was inhibited by triptans [12, 18, 21, 23]. Similar results, with prevention of superficial temporal artery dilation, were reported with the CGRP receptor antagonist olcegepant [24], hence suggesting that the modulation of cranial vasculature tone or perivascular nociception is of paramount importance in migraine treatment, too.

Despite above mentioned evidence, the heated debate about the role of cranial vasculature in migraine pathophysiology is still open, and some authors have questioned whether cranial arteries play a significant role or only represent a negligible epiphenomenon [25, 26]. Even though the precise site where migraine origins still remains elusive, consistent evidence suggests that initial mechanisms may dilate intra- and extra-cerebral arteries, and cranial vasoconstriction may mediate at least a part of the effects of anti-migraine abortive drugs [8]. Thus, considering treatments on the verge of entering the clinical practice, such as CGRP blocking molecules, cranial arteries are undoubtedly of major interest in migraine.

This review reports the effects of triptans and CGRP (receptor) blocking molecules on cranial vasculature in humans, focusing on their specific relevance to migraine treatment. The classification of cranial vessels as intracranial – intracerebral and extracerebral – and extracranial, is shown in Table 1.

**Table 1:** Intracranial intracerebral and extracerebral and extracranial vessels

Cranial vessels may be extracranial and intracranial, and these latter may be distinguished into intracerebral and extracerebral. The middle cerebral artery and the cerebral part of the internal carotid artery are intracerebral vessels, while the cavernous part of the internal carotid artery is extracerebral. On the other hand, according to current imaging detection limitations, both the middle meningeal artery and the superficial temporal artery are considered extracranial vessels. Importantly, the middle meningeal artery has an intracranial and heavily innervated portion that may even be of more relevance in the pathophysiology of migraine than the extracranial portion. However, throughout the text and in accordance with current evidence that are about the extracranial portion, the text refers to middle meningeal artery as to an extracranial vessel.	

### Triptans

The development and consequent introduction of triptans represented an unprecedented revolution in migraine history, being the first successful attempt of rational and mechanism-driven treatment of migraine attacks. Compared to ergot alkaloids (ergotamine, dihydroergotamine and methysergide), that are non-specific serotonin type 1 (5-HT_1_) receptor agonists as they target also 5-HT2, adrenergic and dopaminergic receptors, triptans act as selective agonists at 5-HT_1B_ and 5-HT_1D_ subtypes, displaying a more favourable risk profile to ergots [27, 28].

The rationale for triptans development has been based on the vascular theory of migraine, together with the hypothesis that serotonin and serotonin receptors are involved in migraine pathophysiology. It has been shown that during a migraine attack high levels of hydroxyindoleacetic acid, a serotonin metabolite, are excreted [29] and that monoamine depletors induce migraine attacks that are aborted by intravenous infusion of serotonin [30]. To develop selective cranial vasoconstrictors and to avoid risky side effects of ergot alkaloids (i.e. a marked and long-lasting vasoconstriction in peripheral vessels), Humphrey and colleagues identified the 5-HT1-like receptor, later discovered to consist of both the 5-HT1B and the 5-HT1D receptor subtypes, mostly located in cranial vessels, and then developed the first triptan, known as sumatriptan (GR43175) [31, 32]. Because of its efficacy and safety (including cardiovascular safety), sumatriptan has become a landmark in the treatment of migraine attacks [33]. Nevertheless, some peculiarities, such as the low oral bioavailability and short half-life [34]*,* have favoured the development of new molecules, the so-called “second-generation” triptans (almotriptan, eletriptan, frovatriptan, naratriptan, rizatriptan), with an optimization of the pharmacokinetic profile [35].

Triptans are 5-HT_1B/1D_ receptor agonists, most of them showing a moderate to high affinity for 5-HT_1F_ receptors as well [35]. Immunohistochemical studies have shown that 5-HT_1B_ receptors are mainly located within the smooth muscle and in the endothelium of human middle meningeal [36, 37] and cerebral [38] arteries. Importantly, in in vitro studies, triptans constrict these arteries [37–39]. The 5-HT_1B_ receptors, together with the 5-HT_1D_ and 5-HT_1F_ receptors, are also located within the trigeminal nerve endings and trigeminal nucleus, suggesting that their stimulation could inhibit the release of proinflammatory neuropeptides (e.g. CGRP) and, consequently, the nociceptive transmission [40]. In a randomized placebo-controlled study, the administration of PNU142633, a selective 5-HT_1D_ receptor agonist, failed to alleviate the pain of acute migraine, suggesting a secondary role for 5-HT_1D_ [41]*.* On the other side, selective non-vasoconstrictive 5-HT_1F_ receptor agonists, LY334370 [42] and LY573144 (i.e. lasmiditan) [43] demonstrated clinical efficacy, even though it remains to be confirmed whether these molecules, at therapeutic concentrations, are devoid of any activity on 5-HT_1B_ receptors. Interestingly, lasmiditan did not exer vasoactive effects in supratherapeutic concentrations [44]. Importantly, the high cranial (i.e. middle meningeal artery) 5-HT_1B_ receptor density compared to peripheral (i.e. coronary artery) blood vessels probably renders the triptans relatively selective for producing cranial vasoconstriction [36, 45].

**Fig. 1:**
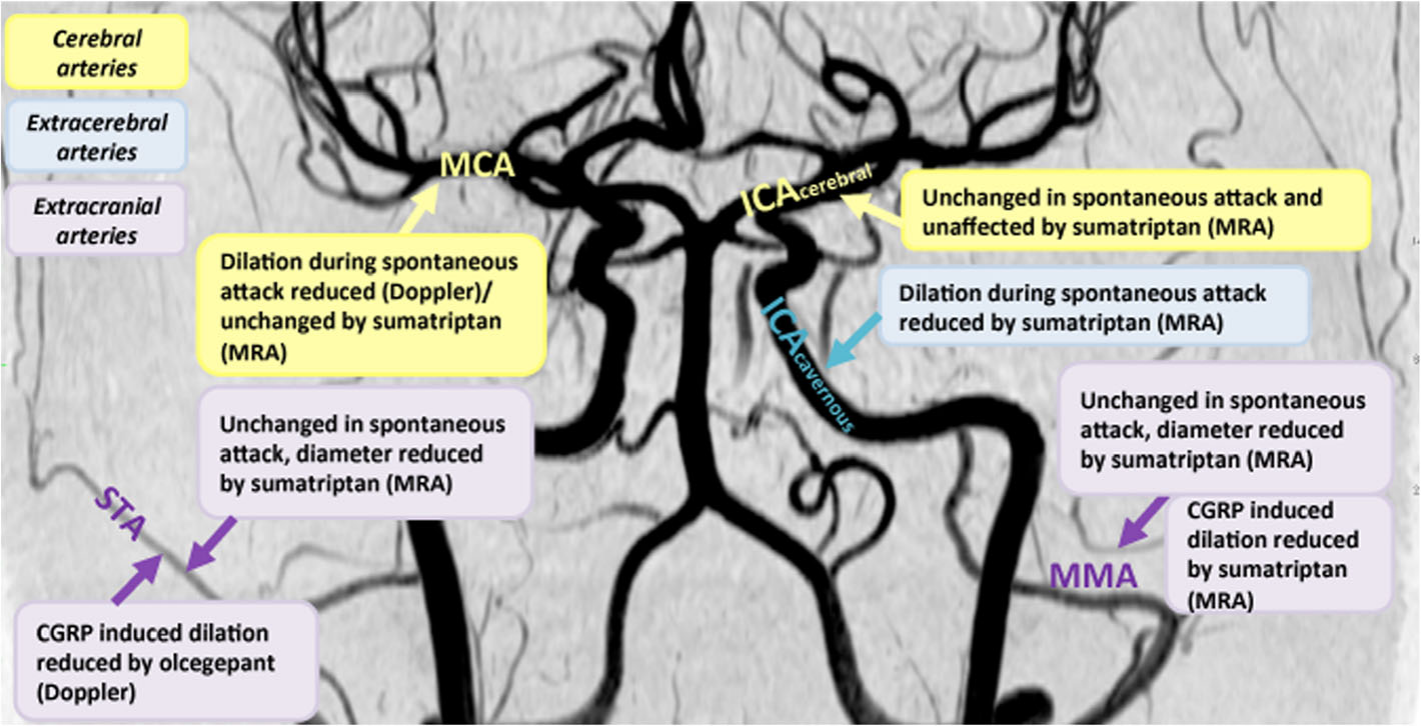
Effects of sumatriptan and olcegepant on cranial vessels in migraine patients. The intracerebral vessels, the middle cerebral artery (MCA) and the cerebral part of the internal carotid artery (ICA_cerebral_), are both shown in yellow. The extracerebral artery, the cavernous part of the internal carotid artery (ICA_cavernous_), is shown in blue. The extracranial vessels, the middle meningeal artery (MMA) and the superficial temporal artery (STA), are both shown in purple. Boxes include description of vessel reactivity during spontaneous and/or CGRP induced attacks as well as vessel response to sumatriptan and/or olcegepant. Imaging modality indicated in parentheses in boxes; magnetic resonance angiography (MRA) or transcranial ultrasound Doppler (Doppler). Image from MRA of healthy volunteer kindly provided by Faisal M Amin

Human experimental data about vessel responses to triptans (Fig. 1) have refined our comprehension of triptan antimigraine effects and, indirectly, of the migraine mechanism. Differently from what observed in vitro and in vivo [46], a placebo-controlled single-photon emission computed tomography (SPECT) study on healthy volunteers showed that sumatriptan infusion did not modify total and regional cerebral perfusion [47]. Interestingly, contrasting data in migraine patients have been initially reported about the correspondence between sumatriptan-related blood velocity modifications, measured by Doppler sonography, and the resolution of migraine attacks [12, 48, 49]. Importantly, combining the measurement of the regional cerebral blood flow and the blood velocity in the middle cerebral arteries, sumatriptan infusion has been shown to reverse the abnormal dilation of the middle cerebral artery in the headache side [12]. This finding suggests that the sumatriptan-induced vasoconstriction occurs only in the dilated vessels, without affecting normal ones.

### Triptans and cranial vasculature

A key feature of migraine is that attacks can be provoked by pharmacological triggers, including GTN [50] and, as detailed below, the provocation migraine models have provided important data on the role of cranial vasculature in migraine.

The efficacy of triptans in GTN-induced headache was investigated in healthy volunteers in double-blind, placebo-controlled, crossover studies. Sumatriptan (6 mg) administered subcutaneously 20 min before GTN (0.12 μg/kg/min) infusion, relieved pain and decreased temporal artery diameter without affecting blood velocity of middle cerebral artery (MCA) [51]. On the other hand, zolmitriptan (5 mg), administered orally during ongoing GTN infusion (0.2 μg/kg/min) had no effects on the induced-headache [52]. Oral triptans (rizatriptan 10 mg, sumatriptan 50 mg and zolmitriptan 2.5 mg) were also tested in migraine patients, in which they have been shown to both decrease diameter and increase resistance of temporal artery, although to a different extent [53]. More recently, the triptan effect was examined after the experimental administration of vasoactive neuropeptides such as CGRP, PACAP-38 and VIP, in healthy volunteers and migraine patients. In a first study, 18 healthy volunteers were randomized to receive an intravenous infusion of human α-CGRP (1.5 μg/min) or placebo, for 20 min [18]. After 45 min, a single dose of subcutaneous sumatriptan (6 mg) was administered to each patient. A high-resolution MRA was performed at baseline, before and after the sumatriptan injection, to measure the changes in the circumference of the MCA and middle meningeal artery (MMA). Compared to placebo, CGRP caused significant dilation of MMA but not of MCA, and sumatriptan reduced the MMA circumference after CGRP pre-treatment by 25% and to a lesser extent on MCA, suggesting that sumatriptan exerts part of its antinociceptive effect primarily acting on MMA. A second study was performed in 24 patients with migraine without aura [23], in which the CGRP intravenous infusion always resulted in a delayed headache, which fulfilled the criteria for migraine-like attacks in 18 patients (75%). MRA was performed in 15 of 18 patients, and of these, 10 (67%) patients reported unilateral head pain. MMA and MCA were dilated only on the painful side. The other 5 patients (33%) reported bilateral head pain, accompanied by a bilateral dilatation of both MMA and MCA. Sumatriptan subcutaneous injection reversed the dilatation of MMA and aborted the migraine attacks, without affecting MCA circumference [23]. These data show that migraine is associated with dilatation of extracerebral and intracerebral arteries, but only the contraction of extracerebral arteries is associated with amelioration of headache.

PACAP-38 is a vasoactive neuropeptide that belongs to the secretin/glucagon/VIP family and it is used to provoke experimental headache and migraine [10]. PACAP-38 is reported to cause delayed headache in healthy volunteers, associated with a significant and long-lasting dilatation of the MMA (up to 23%), but no change in the MCA circumference [19]. In comparison, sumatriptan induced a contraction of the MMA by 12.3% and reversed the delayed headache attack, but no effects on the MCA were observed. The role of PACAP-38 was further investigated in a double-blind crossover study [22] conducted in 22 female migraine patients without aura. Sixteen patients (73%) after PACAP-38 infusion, but only four patients (18%) after VIP infusion (8 pmol/kg/min), reported migraine-like attacks. Both peptides induced a marked dilatation of the extracranial arteries, but *not* of the intracranial arteries. The subcutaneous injection of sumatriptan reversed migraine attacks simultaneously to the constriction of the dilated extracerebral arteries, but *not* the intracerebral arteries.

To date, only one MRA study has explicitly investigated cranial arteries during spontaneous migraine attacks [20]. Migraine attacks, with a median time from pain onset to scan of 5 h 45 min, are not accompanied by extracranial arterial dilation on the pain side, but only by slight dilation of intracerebral arteries, MCA and internal carotid (ICA). In addition, the dilatation of the intracerebral arteries persisted after subcutaneous injection of sumatriptan, which however relieved migraine pain and reduced the circumference of not-dilated extracranial arteries. These data suggested that the vasoconstrictor action of sumatriptan evident in extracranial arteries and in the cavernous portion of the ICA could be relevant to relief migraine. However, these findings do not refuse possible nociceptive input from other extracranial structures, in the absence of dilatation, such as CGRP-releasing sensitized perivascular afferents. Interestingly, recent data in humans suggested that the decrease of capsaicin-induced dermal blood flow may be mediated by the inhibition of CGRP release [54].

### CGRP and cranial vasculature

CGRP is a potent vasodilator expressed and released in the perivascular space by trigeminal sensory neurons with a central role in neurogenic inflammation [55]. CGRP receptor consists of three components: calcitonin-receptor-like receptor (CLR), receptor component protein (RCP) and a specific chaperone called receptor activity modifying protein 1 (RAMP1) [56]. Importantly, CLR and RAMP1 expression has been shown in human middle meningeal [57], middle cerebral, pial and superficial temporal arteries [58], demonstrating the presence of all essential components required for a functional CGRP receptor in these districts*.*

Several small molecule antagonists targeting the CGRP receptor have been developed [59] for the treatment of acute migraine attack and have shown efficacy in clinical trials. Olcegepant (BIBN4096BS) was the first selective and hydrophilic non-peptide CGRP receptor antagonist with an extremely high affinity and specificity for the human CGRP receptor [60] showing clinical efficacy in migraine attacks [61]. In comparison to triptans, which have been extensively studied in humans, most data on vascular effects of “gepants” come from preclinical studies and this has been previously reviewed [62]. Olcegepant, which inhibits dose-dependent relaxation of isolated human cerebral arteries [63], blocks MMA vasodilation following systemic administration of α-CGRP and β-CGRP, without significantly affecting pial artery dilation or the local cortical cerebral blood flow increase [64]. In contrast to the pial vessels, the meningeal arteries have no blood-brain barrier [65], suggesting that olcegepant likely acts outside of the blood-brain barrier [66]*.* In humans, olcegepant per se had no constrictor effect on the middle cerebral, radial, and superficial temporal artery [24], and no influence on global and regional cerebral blood flow [67]. Nevertheless, olcegepant effectively antagonizes the extracerebral vascular effect (e.g. temporal artery dilation) induced by CGRP intravascular administration [24]. A series of orally bioavailable small molecule CGRP receptor antagonists, including MK-0974 (telcagepant), have been then developed giving rise to the pharmacological class of “gepants”. Telcagepant has been shown to be able to abort CGRP-induced vasodilatation on human cerebral and meningeal arteries ex vivo [68]. However, notwithstanding efficacy in clinical trials [69], clinical development of early gepants has been discontinued [70], and accordingly their use in migraine models has been withdrawn.

There are currently four monoclonal antibodies (mAb) in clinical development for migraine prophylaxis: three humanized mAb targeting CGRP (LY2951742/galcanezumab, Eli Lilly and Company; ALD403/eptinezumab, Alder Biopharmaceuticals; and TEV-48215/fremanezumab, TEVA Pharmaceuticals) and one fully human mAb targeting the CGRP receptor (AMG 334/erenumab, Amgen). These biological drugs have shown efficacy, tolerability and few adverse effects in phase 2 randomized control trials [71–76]. However, their exact site and mechanism of action is not completely understood. The new CGRP mAbs are macromolecules (around 150,000 Da) that are unlikely to cross the blood-brain barrier [66]. In line with this, few preclinical studies revealed that humanized CGRP mAb are (i) unable to penetrate the blood-brain barrier in the perfused MCA [77]; (ii) ineffective in inhibiting the responses to CGRP-induced neurogenic vasodilation of the pial artery [78]; and (iii) capable of inhibiting CGRP-induced neurogenic vasodilation of the MMA, which as mentioned above lacks blood-brain barrier [65, 79]. Importantly, it has been recently shown that there is no blood-brain barrier disruption during migraine attacks [80]. All these findings taken into account suggest a peripheral vascular site of action of the mAbs.

A similar alternative under development for the preventive treatment of migraine is blocking CGRP-induced receptor activation through a RNA-Spiegelmer (NOX-C89). This single-strand mirror-image oligonucleotide binds to circulating CGRP and is highly resistant to endogenous nuclease degradation, hence inhibiting its function. Interestingly, this drug could not inhibit neurogenic vasodilation of pial arteries in vivo, which suggested that it is unlikely to penetrate the blood-brain barrier readily [78].

Targeting peripheral CGRP may reduce or prevent the phenomenon (i.e. vasodilation) that has been advocated as a mechanism of headache and associated symptoms, whereas whether long-term inhibition of CGRP outside of the blood-brain barrier induces modulation of central pathways remains unknown. Further studies are needed to fully clarify the exact antimigraine site of action of the CGRP mAbs and NOX-C89.

## Conclusions

From Galen’s quote about meninges and vessels as mediators, together with other structures and mechanisms of migraine pain, long time has passed. In the last years, notwithstanding many detractors, cranial vasculature involvement in the pathogenesis of migraine pain has benefited from experimental data acquired by modern imaging techniques, such as MRA, and specific pharmacological tools, such as triptans. Recently developed human migraine models have suggested that attention should be paid to cranial extracerebral arteries (i.e. MMA) in addition to intracerebral vessels, which were the major focus at the dawn of vascular migraine research. On the basis of current knowledge [20, 23], future studies should investigate whether there are differences in how the perivascular nerves innervate the different sections of the MMA, including dural branches, because it is likely that it is activation or inhibition of the perivascular nerves that is associated with migraine pain relief. In addition, future advanced brain imaging methods will allow to investigate possible dilatation of dural branches of the MMA that are very difficult to visualise with current method, without injection of contrast agents [20]. The availability of innovative migraine-specific drugs, such as CGRP-targeted compounds [69], will further increase our ability to investigate the involvement of cranial vasculature in migraine pain, and will finally allow to properly balance the weight of vessel contribution to the neurovascular theory of migraine.

## Abbreviations

5-HT: serotonin (5-hydroxytriptamine)
CGRP: calcitonin gene-related peptide
CLR: calcitonin-receptor-like receptor
GTN: glyceryl trinitrate
ICA: internal carotid artery
MCA: middle cerebral artery
MMA: middle meningeal artery
MRA: magnetic resonance angiography
PACAP-38: pituitary adenylate cyclase-activating peptide
RAMP1: receptor activity modifying protein 1
RCP: receptor component protein
SPECT: single-photon emission computed tomography
STA: superficial temporal artery
VIP: vasoactive intestinal peptide.
